# Laparoscopic redo pyeloplasty with a buccal mucosal graft

**DOI:** 10.1002/iju5.12567

**Published:** 2022-12-19

**Authors:** Nobuhiko Shimizu, Yukio Naya, Keita Sekine, Kyokushin Hou, Atsushi Okato, Takahito Suyama, Kazuhiro Araki, Hiroshi Masuda, Satoko Kojima

**Affiliations:** ^1^ Department of Urology Teikyo University Chiba Medical Center Ichihara Chiba Japan

**Keywords:** buccal mucosa graft, laparoscopic redo pyeloplasty

## Abstract

**Introduction:**

Redo pyeloplasty can be difficult due to scar tissue or fibrosis. Ureteral reconstruction with a buccal mucosal graft is performed safely and successfully, but most reports of ureteral reconstruction using a buccal mucosal graft are of robot‐assisted surgery, with few reports of laparoscopic‐assisted surgery. A case of laparoscopic‐assisted redo pyeloplasty using a buccal mucosal graft is presented.

**Case presentation:**

A 53‐year‐old woman was diagnosed with ureteropelvic junction obstruction, and a double‐J stent was placed to relieve backache. She visited our hospital 6 months after double‐J stent placement. Three months later, laparoscopic pyeloplasty was performed. At 2 months postoperatively, anatomic stenosis occurred. Holmium laser endoureterotomy and balloon dilation were performed; however, the anatomic stenosis recurred, and laparoscopic redo pyeloplasty with a buccal mucosal graft was performed. After redo pyeloplasty, obstruction was improved, and her symptoms disappeared.

**Conclusion:**

This is the first case of using a buccal mucosal graft for laparoscopic pyeloplasty in Japan.

**Abbreviations & Acronyms**
(99m)Tc‐MAG3: (99m) Tc‐mercaptoacetyltriglycine
BMG: buccal mucosal graft
CT: computed tomography
DJ stent: double‐J stent
LP: laparoscopic pyeloplasty
RP: robot‐assisted laparoscopic pyeloplasty
UPJO: ureteropelvic junction obstruction

**Keynote message**

Redo surgery for UPJO recurrence: it is hard to find healthy and angiogenic tissue due to scar or fibrosis from previous surgery. Recently, redo pyeloplasty using a buccal mucosa graft has been shown to be safe and effective. We performed redo pyeloplasty using a buccal mucosa graft. This is first case in Japan.

## Introduction

Untreated UPJO may lead to hydronephrosis and decreased renal function. Pyeloplasty has been the gold standard treatment for the treatment of UPJO, with a high success rate of greater than 90% in adults.^1^, ^2^ However, in redo surgery for UPJO recurrence, it is hard to find healthy and angiogenic tissue due to scar or fibrosis from previous surgery. Therefore, the success rate of redo surgery is low. Buccal mucosa has been used as a graft in urethral reconstruction with a high success rate. Recently, redo pyeloplasty using a BMG has been shown to be safe and effective.^3^, ^4^ Most reports of ureteral reconstruction using a BMG are of robot‐assisted surgery, and there are few reports of laparoscopic surgery using a BMG. A case of laparoscopic redo pyeloplasty using a BMG is described.

## Case report

**Fig. 1:**
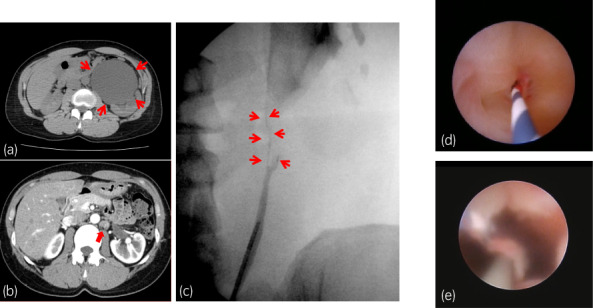
(a) CT shows hydronephrosis (red arrowheads) without crossing vessels when the patient presented with worsening left backache. (b) Contrast‐enhanced CT shows the thickened wall of the UPJ and an increase in the concentration of surrounding fat (arrowheads). (c): Retrograde pyelography when anatomic stenosis occurred showing the stenosis length (white arrowheads). (d) Ureteroscopy shows ureteral stenosis. (e) Ureteroscopy shows improvement of ureteral stenosis after redo pyeloplasty.

**Fig. 2:**
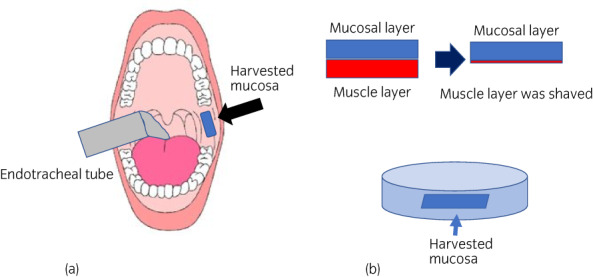
(a) Schema of harvest of the buccal mucosa. The endotracheal tube is secured to the dependent side of the mouth, and the BMG is harvested (black arrow). (b) The graft is shaped off the muscle and placed in saline.

**Fig. 3:**
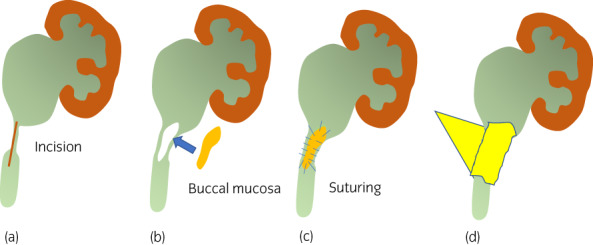
Schema of pyeloplasty using the BMG. (a) A sufficient longitudinal incision on the ureter is made until healthy‐appearing renal pelvis and ureter are seen. (b) The BMG is placed on the incised part. (c) Laparoscopic sewing of the BMG as an anterior onlay graft. (d) The BMG is covered with the omentum.

**Fig. 4:**
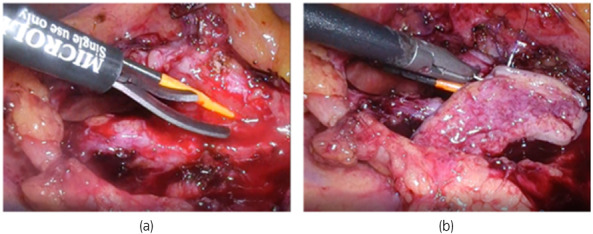
(a) A longitudinal incision of the ureter is made along the anterior surface until healthy‐appearing pelvis and ureter are seen proximally and distally. (b) Laparoscopic sewing of the BMG as an anterior onlay graft.

A 53‐year‐old woman had complained of intermittent left back pain since she was 18 years old. She was diagnosed as having UPJO when she was 38 years old. However, she did not want to be treated and stopped attending a hospital. When she presented again, the left backache was getting worse, and CT showed severe hydronephrosis without crossing vessels, the thickened wall of the UPJ, an increase in the density of surrounding fat, and enlargement of the lymph nodes (Fig. 1a,b). A DJ stent was inserted. Laboratory examination showed a normal estimated glomerular filtration rate (64 mL/min/1.73 m^2^). On (99m)Tc‐MAG3 evaluation, there was normal radioisotope uptake and prolonged time from peak to 50% activity (T (1/2)) in the left kidney (87.27 min). She visited our outpatient clinic 6 months after DJ stenting, and laparoscopic Anderson‐Hynes dismembered pyeloplasty was performed 3 months later. The renal pelvis was tough, with adhesion to the perirenal fat. At 2 months postoperatively, anatomic stenosis occurred, and the length of the stricture was about 2.0 cm (Fig. 1c). Endoscopic techniques, holmium laser endoureterotomy and balloon dilation, were performed; however, after stent removal, left back pain and fever developed, and the anatomic stenosis again recurred. Laparoscopic redo pyeloplasty with a BMG was performed. For the procedure, the endotracheal tube was secured to the dependent side of the mouth, and the BMG was first harvested from the inner cheek by the oral surgeon based on the stenosis length. The graft was shaped off the muscle and placed in saline for later use (Fig. 2). The patient was then placed in a right lateral decubitus position. A camera port was placed in the umbilicus, along with two 5‐mm ports. After medializing colon and visualizing the ureter and renal pelvis, a sufficient longitudinal incision in the anterior wall of the ureter was made until healthy‐appearing pelvis and ureter were seen proximally and distally guiding 6fr straight catheter placed retrograde initial part of surgery (Figs 3a,4a). The 5‐mm port on the left was changed to a 12‐mm port to insert the BMG. The mucosal layer of the graft was oriented toward the lumen and placed on the incised part of the ureter (Fig. 3b). The BMG was anastomosed using 5‐0 absorbable monofilament interrupted suture on the anterior wall (Fig. 3c,4b), and the omentum was sutured over the graft for a blood supply (Fig. 3d). Finally, a 6‐French DJ ureteral stent was placed. Three months after surgery, absence of obstruction was confirmed by ureteroscopy, and the stent was removed (Fig. 1 e). Then, 5 months after surgery, (99m)Tc‐MAG3 evaluation showed significant improvement of (T(1/2)) in the left kidney from 87.27 to 4.75 min, and the patient's main complaint, backache, disappeared.

## Discussion

Reconstruction of long, multifocal ureteral strictures or failed pyeloplasty is a challenging problem. A Boari flap is useful for mid or proximal ureteral strictures, but a flap might not be able to reach the stricture when the bladder capacity is small.^5^ The next surgical option for mid or proximal ureteral strictures is bowel interposition or kidney autotransplantation. Ileal replacement of the ureter has been reported to have a potential risk of infection, ileus, and metabolic acidosis,^6^ and autotransplantation might be a cause of long‐term vascular complications.^7^

Use of a BMG is a gold standard technique for urethral reconstruction. After 2010, several reports of ureteral reconstruction with a BMG showed excellent outcomes in short to intermediate follow‐up.^8^, ^9^ Placing a BMG on an anterior wall is technically easier than placing it on a posterior wall. Rotation of the ureter is necessary for posterior placement. In the present case, anterior placement was performed because the adhesion of the ureter was very strong, and it was hard to rotate the ureter. Vascular support of the BMG is important to prevent graft necrosis. Omental flaps have usually been used. When there is insufficient omentum, Zhao *et al*. reported using perirenal fat.^4^ Recently, Liang *et al*. reported 41 cases of using a lingual mucosal graft instead of a BMG^10^; 40 cases were operated by laparoscopy. In their cases, all patients underwent nephrostomy, and the DJ stent was removed 2 weeks before surgery, reducing ureteral swelling. In the present case, the DJ stent was placed 9 months before the first pyeloplasty and ureteral swelling and adhesion was severe. It might be one of the causes of the failure of the first operation. It is important to consider whether LP or RP is better for the first UPJO. A meta‐analysis of LP and RP showed similar success rates of over 95% in both groups.^11^ Lucas *et al*. reported that there was no difference between LP and RP in freedom from secondary surgery on multivariate analysis.^12^ Thus, LP is not inferior to RP.

In our view, reconstruction of ureteral strictures using BMGs seems to be sufficiently feasible with conventional laparoscopic procedures without robot assistance.

In conclusion, this is the first case in which a BMG was used for laparoscopic reconstruction of a ureter in Japan.

## Author contributions

**Nobuhiko Shimizu:** Writing – original draft. **Yukio Naya:** Conceptualization; supervision. **Keita Sekine:** Data curation; writing – review and editing. **Kyokushin Hou:** Writing – review and editing. **Atsushi Okato:** Resources; writing – review and editing. **Takahito Suyama:** Supervision; writing – review and editing. **Kazuhiro Araki:** Supervision; writing – review and editing. **Hiroshi Masuda:** Supervision; writing – review and editing. **Satoko Kojima:** Supervision; writing – review and editing.

## Conflicts of interest

The authors declare no conflict of interest.

## Approval of the research protocol by an Institutional Reviewer Board

Not applicable.

## Informed consent

Not applicable.

## Registry and the Registration No. of the study/trial

Not applicable.

## Data Availability

The data that support the findings of this case report are available from the corresponding author, Yukio Naya, on reasonable request.
